# Design of a randomized controlled trial of Internet-based cognitive behavioral therapy for treatment-induced menopausal symptoms in breast cancer survivors

**DOI:** 10.1186/s12885-016-2946-1

**Published:** 2016-11-25

**Authors:** Vera Atema, Marieke van Leeuwen, Hester S. A. Oldenburg, Valesca Retèl, Marc van Beurden, Myra S. Hunter, Neil K. Aaronson

**Affiliations:** 1 Division of Psychosocial Research and Epidemiology, The Netherlands Cancer Institute, Plesmanlaan 121, 1066 CX Amsterdam, The Netherlands; 2 Department of Surgical Oncology, The Netherlands Cancer Institute, Plesmanlaan 121, 1066 CX Amsterdam, The Netherlands; 3 Department of HTSR, School of Governance and Management, University of Twente, PO Box 217, 7500 AE Enschede, The Netherlands; 4 Department of Gynecology, The Netherlands Cancer Institute, Plesmanlaan 121, 1066 CX Amsterdam, The Netherlands; 5 Department of Psychology (at Guy’s), Institute of Psychiatry, Kings College London, 5th floor Bermondsey Wing, Guy’s Campus, SE1 9RT London, UK

**Keywords:** Breast cancer, Menopause, Hot flushes, Cognitive behavioral therapy, Internet-based, Self-management, eHealth, Randomized controlled trial, Cost-effectiveness

## Abstract

**Background:**

Menopausal symptoms are common and may be particularly severe in younger women who undergo treatment-induced menopause. Medications to reduce menopausal symptoms are either contra-indicated or have bothersome side effects. Previous studies have demonstrated that face-to-face cognitive behavioral therapy (CBT) is effective in alleviating menopausal symptoms in women with breast cancer. However, compliance with face-to-face CBT programs can be problematic. A promising approach is to use the Internet to make this form of CBT more accessible and feasible for patients. This study is evaluating the efficacy and cost-effectiveness of an Internet-based CBT program, with or without therapist guidance, in alleviating or reducing the severity of menopausal symptoms.

**Methods/design:**

In a multicenter, randomized controlled trial we are evaluating the efficacy of two Internet-based CBT programs in alleviating or reducing the impact of menopausal symptoms, and particularly hot flushes and night sweats, in breast cancer survivors who have experienced a treatment-induced menopause. Secondary outcomes include sexual functioning, sleep quality, hot flush frequency, psychological distress, health-related quality of life and cost-effectiveness. We will recruit 248 women who will be randomized to either a therapist guided or a self-management version of the 6-week Internet-based CBT program, or to a usual care, waiting list control group. Self-administered questionnaires are completed at baseline (T0), and at 10 weeks (T1) and 24 weeks (T2) post-randomization.

**Discussion:**

Internet-based CBT is a potentially useful treatment for reducing menopausal symptoms in breast cancer survivors. This study will provide evidence on the efficacy and cost-effectiveness of such an Internet-based CBT program, with or without therapist support. If demonstrated to be efficacious and cost-effective, the availability of such structured supportive intervention programs will be a welcome addition to standard medical treatment offered to cancer patients with treatment-induced menopause.

**Trial registration:**

The study is retrospectively registered at ClinicalTrials.gov on January 26th 2016 (NCT02672189).

## Background

Breast cancer is the most common cancer among women worldwide, with approximately 1,7 million new cases reported in 2012 [1]. Due to increasing numbers of patients with cancer and improving survival rates [2] more interest and research has focused on health-related quality of life (HRQOL) of breast cancer survivors, including treatment-induced menopausal symptoms. Nearly 30% of all women with breast cancer are premenopausal at time of diagnosis [3]. Breast cancer treatment, including chemotherapy and endocrine treatment induce premature menopause, either by damaging the ovaries or altering the uptake of estrogen [4, 5]. Oophorectomy also results in surgically induced menopause [6, 7].

Premature menopause is a major concern of younger women undergoing treatment for breast cancer [8]. Primary menopausal symptoms include hot flushes, night sweats, vaginal dryness, decreased libido, dysuria and urinary incontinence. Secondary symptoms include insomnia due to night sweats, dyspareunia because of vaginal dryness, weight gain, and psychological distress [4, 9, 10]. Among these menopausal symptoms, hot flushes are considered to be the most disruptive, with prevalence rates between 63 and 80% in breast cancer survivors [9, 11–14]. Hot flushes are often more severe in women who experience treatment-induced menopause, compared to women going through natural menopause [7, 15, 16]. The exact etiology of hot flushes is not fully understood. They appear to be the result of a dysfunction in the thermoregulatory system via the hypothalamus, due to (natural or treatment-induced) changes in estrogen levels. Together with changes in the neurotransmitters serotonin and norepinephrine they impact the thermoregulatory homeostasis [17–19].

Menopausal symptoms are an important source of morbidity [20] and discomfort [9, 10] in breast cancer patients and survivors, and they may also adversely affect women’s sexual functioning and overall HRQOL [8, 21–26]. Moreover, menopausal symptoms are an important reason why some women discontinue endocrine treatment [27–29]. Many women experiencing treatment-induced menopause report unmet needs for information about how to manage menopausal symptoms [30].

Menopausal symptoms can be treated medically by either hormone replacement therapy (HRT) or non- hormonal treatment modalities including clonidine, selective serotonin reuptake inhibitors (SSRI’s) and gabapentin [31, 32]. Although highly effective in alleviating menopausal symptoms, HRT is contra-indicated in women with a history of breast cancer [33, 34]. Non-hormonal treatments are moderately effective but have a range of common and bothersome side-effects [35–39]. Many breast cancer survivors with treatment-induced menopause prefer non-medical treatments for their menopausal symptoms [14].

There is increasing evidence that behavioral interventions have a positive impact on symptoms experienced by women with naturally occurring and treatment-induced menopause [40–46]. Cognitive behavioral therapy (CBT) is the only type of behavioral intervention with level 1 efficacy evidence for both women with naturally occurring and treatment-induced menopause. Use of CBT has been recommended by the North American Menopause Society [47].

Hunter and colleagues [48] developed a cognitive model of menopausal hot flushes to explain symptom perception, cognitive appraisal, and behavioral reactions to symptoms. Based on this model, they developed a form of CBT, including relaxation and psycho-education, that focuses on the relationships between thoughts, feelings and behavior [49]. Their CBT intervention incorporates information about symptoms, monitoring and modifying precipitants, relaxation and stress management, cognitive restructuring of unhelpful assumptions and automatic thoughts, and encouraging helpful behavioral strategies [40].

Three randomized controlled trials (RCT’s) have demonstrated the efficacy of this CBT program in reducing menopausal complaints in women from the general population [42] and in women with menopausal symptoms after breast cancer treatment [43, 44]. In the study of Ayers and colleagues [42], 140 women from the general population were randomly assigned to group CBT, guided self-help CBT or a no treatment control group. Both CBT groups decreased significantly in hot flush and night sweat (HF/NS) problem ratings compared to the control group 6 weeks and 6 months after randomization. These findings in the general population, however, cannot necessarily be generalized to cancer patients. Women who go through natural menopause have much greater opportunities to communicate with and relate to their same-aged peers who have gone through or are going through menopause. Cancer patients distinguish themselves from women who go through natural menopause by the severity of their symptoms, and the typically younger age at which they experience treatment-induced menopause.

In the study of Mann et al. [43] 96 breast cancer survivors with problematic menopausal HF/NS were randomly assigned to either group CBT or usual care. In this study, women who had received CBT had a significantly reduced HF/NS problem rating as compared to the control group at 9 weeks and at 6 months post-randomization. Our group [44] conducted a 4-group RCT (the EVA trial) to evaluate the efficacy of group CBT, physical exercise (PE), or a combination of CBT and physical exercise in alleviating treatment-induced menopausal symptoms in breast cancer survivors, as compared to a waiting list control group (WLC) (*N* = 422). All intervention groups reported a significant decrease in levels of endocrine symptoms 12 weeks and 6 months after randomization, compared to the usual care waiting list control group. The two groups that included CBT also reported a significant decrease in HF/NS problem rating at 12 weeks and 6 months. However, in the RCT of Duijts and colleagues, noteworthy problems were observed in compliance with the group CBT. More than 50% of women attended less than 4 of the 6 CBT sessions. Many women reported scheduling conflicts related to work and child care as the reason for their under-compliance. Per-protocol analysis suggested that, if compliance rates could be increased, the intervention would be even more effective. Many women indicated an interest and willingness to undergo a CBT program administered via the Internet. This was viewed as a more flexible alternative to face-to-face group CBT, and thus is hypothesized to result in increased compliance and increased efficacy of the treatment. Further, two-thirds of breast cancer patients believe that Internet-based therapy is equally or more likely to result in improved physical and mental health, as compared to face-to-face therapy [50].

A cost-effectiveness analysis (CEA) of the EVA-trial data showed that CBT was likely to be the most cost-effective intervention, with incremental cost-utility ratios of €22,502/quality adjusted life year (QALY) for CBT versus WLC and €28,078/QALY for PE versus WLC [51]. Providing CBT via an Internet-based platform, either in a guided or self-managed format may further increase the cost-effectiveness of this intervention.

There is growing evidence that Internet-based CBT is an effective method to treat a range of psychosocial problems in both the general population and in cancer survivors [52–56]. The overall mean effect size of (ES) of Internet-based therapy is 0.53 which is comparable to the average ES of traditional face-to-face therapy [57, 58]. In general, Internet-based interventions with therapist guidance have been found to be more effective then Internet-based interventions without any therapist guidance [53, 54, 56]. However, self-management interventions have clear benefits in terms of accessibility and convenience, and lower costs. For these reasons, increasing attention is being paid to optimizing self-management variants of CBT programs and to identifying who may benefit most from them [59–62]. For example, it has been argued that self-managed interventions are more effective with motivated patients who have moderate rather than severe symptoms [63, 64].

Self-managed interventions are often associated with low compliance rates. However, this can be improved when the self-management interventions include prior screening and are part of a ‘closed system’ (i.e. not accessible without some form of eligibility check) [59–61]. Under such conditions, compliance rates are similar to those found in face-to face therapy [60]. There is also evidence that compliance in self-management interventions can be further increased by the use of reminders [65–67].

### Current study

This randomized, controlled, multicenter trial, “EVA-Online”, is designed to evaluate the efficacy and cost-effectiveness of two Internet-based CBT programs, one guided and the other self-managed, to reduce or ameliorate treatment-induced menopausal symptoms in women who have had breast cancer. We hypothesize that women in both Internet-based CBT groups will report a significantly greater reduction in overall levels of menopausal symptoms and/or HF/NS problem rating than women in the control group. Secondarily, we hypothesize that women in both Internet-based CBT groups will report significantly greater improvement in sexual functioning, sleep quality, hot flush frequency, psychological distress and HRQOL than women in the control group. We will also evaluate the relative efficacy of self-managed versus guided Internet-based CBT, but this will be done in a more descriptive manner, given that the trial is not powered to test these differences formally. We are also investigating, in a more exploratory manner, the extent to which program compliance serves as a moderator, and hot flush beliefs and behavior as mediators of the treatment effects on the primary outcomes of interest [68]. Finally, we hypothesize that both internet-based CBT groups have a higher probability of being cost-effective compared to the control group. If demonstrated to be efficacious and cost-effective, the availability of such structured supportive intervention programs will be a welcome addition to standard medical treatment offered to cancer survivors with treatment-induced menopause.

## Methods

**Fig. 1:**
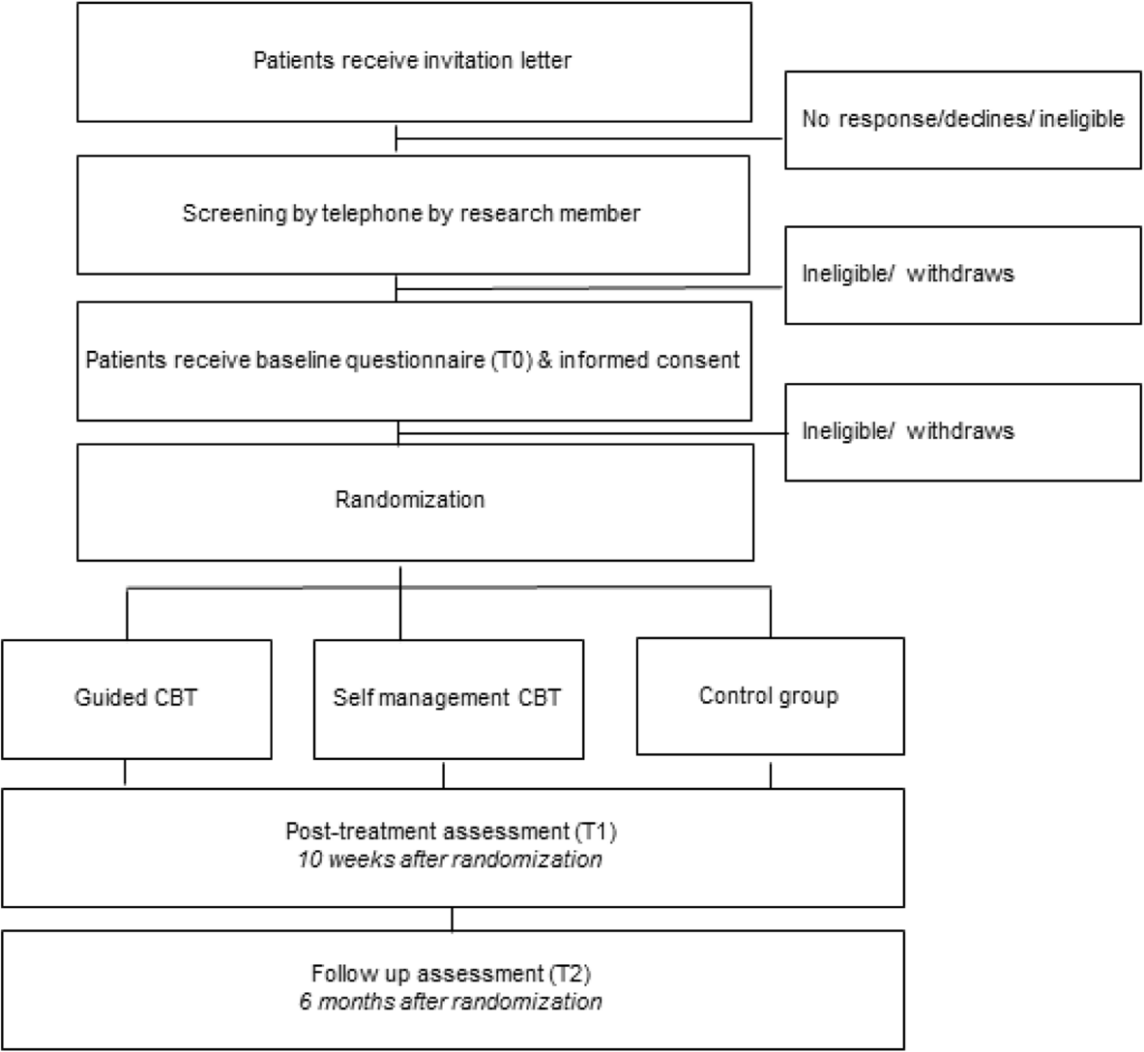
Overview of the overall trial design

In this trial, patients are randomized to one of three study arms. There are two interventions arms, i.e. Internet-based guided CBT and Internet-based self-management CBT, and one waiting list control arm. The design of the trial and the anticipated flow of the participants are displayed in Fig. 1. This trial protocol (June 25^th^, 2015, version 2) has been approved by the Institutional Review Board (IRB) of The Netherlands Cancer Institute (under number NL 53182.031.15), as well as by the review boards of all hospitals from which patients are being recruited. Any important protocol modifications (not anticipated) will be reported to the IRB and the trail registration (clinicaltrials.gov).

### Study sample

The study sample will be composed of 248 women, 50 years of age or younger at time of diagnosis, with histologically confirmed primary breast cancer (stages: T1 – T4, N0 – N3 and M0). All women will have been premenopausal at the time of diagnosis, and will have experienced a treatment-induced menopause due to (neo) adjuvant chemotherapy and/or hormonal therapy and/or oophorectomy. In case of treatment-induced menopause due to (neo) adjuvant chemotherapy or oophorectomy, treatment should have been completed a minimum of 4 months and a maximum of 5 years prior to study entry (with the exception of Herceptin use). Women may currently be receiving adjuvant hormonal therapy. All women should be disease-free at time of study entry. Potentially eligible women are screened for the presence of problematic HF/NS during the past 2 months. They must have experienced at least ten hot flushes or night sweats during the past week and these HF/NS should be experienced as problematic (as indicated by an average score of 2 or higher on three items of the Hot Flush Rating Scale ([69]).

Women are excluded from the study if they lack basic proficiency in Dutch, have been treated in the past for another form of cancer (other than basal cell carcinoma), have serious overt cognitive or psychiatric problems that would preclude them from following the intervention or completing the study questionnaires, or have no Internet access. Patients participating in concurrent studies or rehabilitation programs focused on the reduction of or coping with menopausal symptoms (i.e. relaxation, mindfulness, psychoeducation and/or CBT) are also excluded.

### Recruitment and randomization

We are recruiting patients from 13 community and university hospitals in the Netherlands. We identify potentially eligible patients through hospital registries and the database of the Netherlands Cancer Registry. Potentially eligible patients are sent a personalized letter by their treating physician informing them about the study and the Internet-based program. Women are asked to respond to this invitation by returning either a short screening questionnaire (when there is interest in participating), or a postcard indicating reasons for declining participation. Non-respondents are sent a reminder 2 weeks after the first invitation.

The women who express interest in the study, but do not meet the eligibility criteria receive a personalized letter that explains why they are not eligible. When initial eligibility criteria are met, women are contacted by telephone to confirm their eligibility, to explain the Internet-based program and the RCT, and to confirm their willingness to invest the requisite time and effort if they are randomized to either of the two intervention groups. A baseline questionnaire and informed consent form are sent to eligible and motivated patients. Upon return of both to the study staff, patients are randomized to the guided Internet-based CBT group (*n* = 83) the self-management Internet-based CBT group (*n* = 83) or to a usual care, ‘waiting-list’ control group (*n* = 82) using the minimization technique [70] with age (<40 years; 40–45 years, >45 years), current endocrine treatment for breast cancer (yes; no), time since chemotherapy (<1 year; 1–3 years; > 3 years) and current use of non-hormonal treatments for hot flushes (antidepressants, clonidine, gabapentin) (yes; no) as stratification variables. Due to the nature of the study, blinding is not possible.

### Study arms

All participants randomized to either of the two intervention groups receive access to the same Internet-based CBT program. The primary focus of the program is on HF/NS, with participants being encouraged to develop helpful cognitive and behavioral coping styles. Other problem areas, including sexuality, weight gain and stress are also addressed by the program. The CBT program, is based on the work of Hunter and colleagues [40, 42, 49, 71] and has been tailored for use by breast cancer survivors [43, 44]. The program consists of six modules, which preferably should be followed in six consecutive weeks (for a description of the program see Table 1). Each module contains the following sections: reflection on progress in past week, an introduction, psycho-education, and in-session and homework assignments. Video clips of experts (a breast surgeon and a sexologist) provide complementary information to that presented in the written text. Also incorporated in the program are testimonials (short video clips and written text) of women who have gone through treatment-induced menopause and who have followed the program. The written text also provides examples of how to complete the homework assignments. The six modules are presented in a sequential order, wherein each module builds upon the previous one. The average estimated time investment is 1 h a week to complete a module and 30 min per day for homework (e.g. keeping a daily hot flush/night sweats diary and relaxation exercises).

**Table 1:** Description of Program Modules

Module 1 Welcome	▪ Introduction to the online program
	▪ Psycho-education about the effect of breast cancer on menopause, menopausal symptoms and the influence of relaxation.
	▪ In-session assignment: making a schedule for reading the sessions and doing homework
	▪ Homework: keeping a hot flushes and night sweats diary; practicing relaxation techniques
Module 2 Hot flushes	▪ Psycho-education about the physiology of HF/NS and the role of thoughts, feelings and behaviors
	▪ In-session assignment: recognizing patterns of and triggers for hot flushes; cognitive restructuring of unhelpful thoughts
	▪ Homework: as before + monitoring triggers and applying helpful thoughts
Module 3 From stressing to relaxing	▪ Psycho-education about stress, the relationship between stress and hot flushes, cognitive and behavioral stress management techniques, relaxation.
	▪ In-session assignment: identification of stressful events, usual reaction to stress and goal setting to reduce stress
	▪ Homework: as before + implementation of stress goal
Module 4 Improving sleep	▪ Psycho-education about sleep, sleeping problems and how to improve quality of sleep, cognitive and behavioral reactions to sleep problems/night sweats.
	▪ In-session assignment: sleep hygiene questionnaire, goal setting to improve sleep.
	▪ Homework: as before + implementation of sleeping goals
Module 5 My body and sexuality	▪ Psycho-education about sexual problems and weight issues, cognitive and behavioral precipitants and consequences of sexual problems and weight issues.
	▪ In-session assignment: goal setting for sexual problems (if present) and weight issues (if present)
	▪ Homework: as before + implementation of goals
Module 6 Keep progressing	▪ Psycho-education about the (benefits of) using an action plan.
	▪ In-session assignment: identification of helpful cognitive and/or behavioral strategies as discussed/learned throughout each module, goal setting for maintenance plan; identification of possible barriers and how to overcome them.
	▪ Homework: as before + implementation of maintenance plan

#### Intervention group: guided Internet based CBT program

The women in the guided Internet-based CBT group receive, in addition to the online CBT program, a scheduled 30 min telephone interview prior to the start of the program and weekly feedback per email during the course of the program. After receiving and reading the weekly feedback, participants are given access to the next module. The interview and weekly feedback are provided by a trained therapist (medical social worker or psychologist). The therapist has access to the participants’ in-session reflection, homework assignments and daily hot flush diary. Participants can also contact the therapist by email if they have any questions. Monitoring of the integrity of the intervention is carried out at regular intervals by the study coordinator.

#### Intervention group: self-management Internet-based CBT program

The women in the self-management Internet-based CBT group have access to the online CBT program as described above and receive weekly reminders by email. Participants in this group have access to the entire CBT program from time of enrollment forward, but are advised and encouraged to follow the program using a weekly schedule for 6 consecutive weeks.

#### Waiting list control group

Participants in the waiting list control group are offered the opportunity to follow the CBT program after completion of the 6-month follow up questionnaire. There are no other behavioral interventions for menopausal symptoms available through the participating hospitals, but women in all groups are asked at the follow-up assessments if they engaged in any other means to reduce their menopausal symptoms.

### Data collection

All trial participants complete a battery of self-report questionnaires at equivalent points in time: T0 (at baseline prior to randomization), T1 (10 weeks after randomization) and T2 (6 months after randomization). A reminder is sent to participants who do not return the questionnaire within 1 week. If a woman does not complete the questionnaire in the week after the reminder, she is contacted by telephone. Every effort is made to obtain a final post-intervention assessment for patients who discontinue the intervention. Additionally, all women, including those in the control group, are asked if, during the period of the study, they had engaged in any (other) activities to alleviate their menopausal symptoms (e.g., contacts with patient self-help groups, use of Internet resources, alternative remedies, etc.).

### Study measures

#### Sociodemographic and clinical data

The patients’ age, education, marital status, living situation, work status, weight and height, medication use (including alternative medications or therapies for menopausal symptoms) and life style variables (e.g. smoking, physical activity/exercise) are obtained via the baseline questionnaire. Clinical information, including date of diagnosis, tumor characteristics, and treatment history are abstracted from the patients’ medical records and via self-report. During the follow up period, participants are asked if they had resumed menstruation and whether they had discontinued endocrine treatment, if applicable.

#### Outcome measures

A detailed description of the outcome measures is provided in Table 2. Briefly, the primary outcome measures include standardized self-report questionnaires assessing hot flushes and night sweats problem rating using the Hot Flush Problem Rating Scale (HFRS) [69], and overall levels of menopausal symptoms using the Functional Assessment of Cancer Therapy –Endocrine symptom scale (FACT-ES) [72]. Secondary outcome measures include standardized self-report questionnaires assessing sexual functioning (SAQ) [73], sleep quality (GSQS) [74]; hot flush frequency (HFRS) [69], psychological distress (HADS) [75, 76] and health-related quality of life (SF-36 Health Survey) [77, 78].

**Table 2:** Study outcome measures and corresponding questionnaires

Variable	Questionnaire	Details
Primary outcomes		
Hot flush/Night sweats problem rating	HFRS	▪ 3 items (subscale), 10 point scale
		▪ Score range: 0–10 (mean scores are used); higher scores indicate higher problem rating
		▪ Time frame: 1 week
		▪ Test-retest reliability 0.80
Overall level of menopausal symptoms	FACT-ES	▪ 18 items, 4 point Likert scale
		▪ Score range: 0–72; higher scores indicate fewer menopausal symptoms
		▪ Time frame: 1 week
		▪ Cronbach’s alpha: >0.80
Secondary outcomes		
Sexual functioning	SAQ	▪ 10 items, 4 point Likert scale
		▪ Subscales: pleasure; discomfort; habit
		▪ Score range: pleasure 0–18 higher scores indicate higher levels of pleasure; discomfort 0–6 lower scores indicates lower levels of discomfort; habit 0–3; single item (0 ‘less sexual activity than usual’ to 3 ‘much more sexual activity than usual’
		▪ Time frame: past month
		▪ Test-retest kappa: 0.50–0.76
Sleep quality	GSQS	▪ 14 items, dichotomous (yes/no) scale
		▪ Score range: 0–14; higher scores indicate more sleep problems
		▪ Time frame: past month
Hot flush frequency	HFRS	▪ 2 items (subscale); open-ended frequency scale
		▪ Score range: reported average of HF/NS per week
		▪ Time frame: past week
		▪ Test-retest reliability 0.80
Psychological distress	HADS	▪ 14 items, 4-point Likert scale
		▪ Subscales: depression (HADS-D); anxiety (HADS-A)
		▪ Score range: total score 0–42; subscale scores 0–21 higher score indicates more psychological distress
		▪ Time frame: past week
		▪ Cronbach’s alpha: HADS-D 0.67–0.90; HADS-A 0.68–0.93
Health-related quality of life	SF-36	▪ 36 items, dichotomous and 3- to 6-point Likert scales
		▪ Subscales: physical functioning, role limitations due to physical health problems, bodily pain, social functioning, general mental health, role limitations due to emotional problems, vitality, general health perceptions
		▪ Score range: 0–100; higher score indicates higher levels of functioning/well-being
		▪ Time frame: past week
		▪ Cronbach’s alpha: 0.66–0.91 (mean 0.84)
		▪ For the cost-effectiveness analysis we will map the SF-36 onto the EuroQol5D to obtain utilities

### Moderating and process measures

#### Hot flush beliefs and behaviors

Beliefs about hot flushes and night sweats are considered moderators of the effect of the CBT program [79]. These are assessed with the Behavior Short Form HFNS Beliefs and Behavior Scale, a 16 item scale that includes items from the Hot Flush Beliefs Scale [80] and the Hot Flush Behavior Scale [81], (Hunter, personal communication).

#### Compliance with the intervention

Women are asked to indicate the number of CBT program modules they completed, the frequency with which they did the homework assignments and the total amount of time (in weeks) that they used the program. We are also able to monitor the actual use (frequency and duration) of the online CBT program through log data. We consider completion of the first three modules evidence of an acceptable level of program compliance because these are the modules that specifically focus on the primary outcomes.

Women who do not complete the intervention are asked to indicate their reason(s) for discontinuation (e.g., lack of motivation, illness, program burden). We assess general self-efficacy, social support and intention to complete the program as potential predictors of compliance. General self-efficacy is measured with the General Self-Efficacy scale from [82]. It includes 10 items (4 point Likert scale). Social support is assessed by the emotional/informational support subscale (8 items Cronbach’s alpha 0.96) of the Medical Outcome Study- Social Support Scale ((MOS-SS [83]). Behavioral intention is assessed by three items (5 point Likert scale) derived from the Theory of Planned Behavior [84].

#### Patients’ evaluation of the intervention program

At T1 (immediate post-intervention), women in both intervention groups are asked to complete a short questionnaire about their experience with the Internet-based CBT program. This will include questions about the perceived efficacy of and satisfaction with the program, whether they would suggest any changes to the program, and if they would recommend it to other women experiencing treatment-induced menopausal symptoms. In addition, in an effort to better understand how the program might be improved, we will conduct telephone interviews (30 min) after the T2 assessment with women who indicated on the questionnaire that the intervention did not have the desired effect and/or gave the intervention a low rating and/or would not recommend the program to others.

### Cost effectiveness

We will perform a cost-effectiveness analysis (CEA) using a validated health economic model as developed for use earlier in the EVA-trial [51]. The cost-effectiveness of the Internet-based guided CBT versus the Internet-based self-management CBT versus usual care will be expressed as: (1) cost per clinically relevant significant reduction on the problem-rating scale of the HFRS and (2) cost per QALY gained. A change of at least two points on the ten-point problem-rating scale of the HFRS, and of 0.5 SD on the FACT-ES is considered a relevant improvement [49]. A societal and hospital perspective from the Netherlands, plus a 5 year time horizon will be adopted. Future costs and effects will be discounted at 4 and 1.5%, respectively, according to the Dutch guidelines. A Markov model will be constructed with 3 mutually exclusive health states: “menopausal symptoms”, “reduction in menopausal symptoms”, and “recurrence”. Using a 6-month cycle length, the model will simulate the course of events in a hypothetical cohort of 1000 breast cancer survivors. The “effectiveness” part of the cost-effectiveness equation will be based on the HFRS (1) and SF-6D (2). The SF-6D [85], derived from the SF-36, will be mapped onto the EuroQol-5D, which will provide utilities. With the utilities, the SF-6D allows indirectly generating quality adjusted life years (QALYs) to be used in cost-effectiveness analysis.

For the direct costs, we will ask all women to report at T1 and T2 their use of health care services (e.g., GP, medical specialist, paramedical care etc.), medication use and workdays lost due to illness. In calculating the intervention costs, we will include the time spent by health professionals in providing feedback to participants (where applicable), staff training, administration, and material costs. Detailed descriptions of the intervention will be made to identify specific cost items and corresponding volumes of resource use. Subsequently, costs will be calculated by multiplying unit prices (or appropriate tariffs) by volumes of use, following the Dutch pharmacoeconomics costing guidelines [86].

The indirect costs will be measured by the Friction cost method, which is the period over which the production loss is calculated, i.e. the time that an employer needs to replace a sick employee. The calculation of the average labor costs per working day will be based on the weighted average labor costs of full-time and part-time employed persons in the Netherlands [87].

### Power calculation

The HFRS problem rating scale and the FACT-ES scale score, assessing endocrine symptoms, are the primary outcomes on which sample size calculations are based. With a total sample of 198 women (66 per group), and under the assumption of no interaction, the study will have 80% power to detect a 0.5 standard deviation difference (Cohen’s effect size) with a p value of 0.05 (two sided test) [88]. We anticipate that this effect size will be sufficient to demonstrate the efficacy of the interventions, as the CBT group intervention in the EVA-study yielded effect sizes for the primary outcomes of approximately 0.5 [44]. We will recruit 248 women into the study, to allow for an attrition rate of approximately 20% (i.e. women who discontinue participation in the study entirely, including failure to complete follow up questionnaires). Women who discontinue participation in one of the intervention groups but complete the follow up assessments will be included in the analysis.

### Statistical analysis

All data will be anonymized prior to final data analysis. The data set will not contain any personal identifiers. Only study staff will have access to these data.

Analyses will first be performed to evaluate the comparability of the intervention groups (guided versus self-management) and control group at baseline in terms of sociodemographic and clinical characteristics. ANOVA tests or appropriate non-parametric statistics will be used, depending on the level of measurement. If, despite the stratified randomization procedures, the groups are found not to be comparable on one more background variables, those variables will be employed routinely as covariates in subsequent analyses. Questionnaire scores will be calculated according to published scoring algorithms. We will compare both intervention groups with the control group over time using multilevel procedures with repeated measures, using a restricted maximum likelihood (REML) solution to model specific contrasts between groups and follow-up assessment [89]. Within each multilevel model the control group will be the reference category. For the analysis of the secondary outcome measures, appropriate statistical adjustments will be made for multiple testing. Differences in mean change scores over time between the intervention groups and the control group will be accompanied by effect sizes (ES). These effect sizes will be calculated using standard statistical procedures. Effect sizes of approximately 0.5 are considered clinically significant [90]. All analyses will be conducted on an intention to treat (ITT) basis. In addition, per-protocol (PP) analyses will be performed (as a secondary analysis) on patients who met criteria for minimal compliance with the intervention(s). Supplementary analyses will be carried out in which data relating to compliance with the program elements will be taken into account. Specifically, we will determine whether the level of compliance is associated significantly with the change over time in the primary and secondary outcomes. We will also investigate whether program effectiveness varies significantly as a function of changes in hot flush beliefs and behaviors.

#### Cost-effectiveness analysis

We will use a Markov model to perform an incremental cost-effectiveness and cost-utility analyses. The cost-effectiveness ratio is calculated by dividing the difference between the mean total costs of the intervention and control groups by the difference in mean primary clinical effects of the groups [51]. The incremental cost-utility ratio expresses the additional costs of the intervention per quality-adjusted life year (QALY) gained, compared to the usual care group.

#### Modeling statistics

State of the art health economic methods will be applied. These include the estimation of the degree of uncertainty about each input parameter and the use of probabilistic sensitivity analyses. Parameter values will be drawn randomly from the assigned distributions, using Monte Carlo simulation with 10,000 iterations. The degree of uncertainty will be illustrated by using confidence intervals for costs and health effects. Scatter-plots, confidence ellipses on cost-effectiveness planes and cost-effectiveness acceptability curves will be presented [91–93]. We will use the European informal ceiling ratio of €30,000 per QALY [86]. Finally, a Budget Impact Analysis will be performed from the perspective of the health care provider.

All study results will be published in peer-reviewed publications and will result in a Ph.D. thesis. Authorship eligibility will be based on the Vancouver Protocol [94]. Participating patients will receive a lay summary of the results.

## Discussion

A relatively large percentage of young breast cancer survivors experience treatment-induced menopausal symptoms, with hot flushes being the most common and severe symptom [9, 11–14]. There is a need for effective and safe non-medical treatment options for these symptoms. Studies show that, both in the general population and among breast cancer survivors, CBT is an effective treatment method for alleviating menopausal symptoms when provided in a group setting or through guided self-help [42–44, 49]. However, compliance can be problematic [44]. A promising approach is to make this form of CBT more accessible and feasible for participants by administering it via the Internet. In the current trial we are evaluating the efficacy and cost-effectiveness of Internet-based CBT in alleviating or reducing menopausal symptoms and HF/NS problem ratings in younger breast cancer survivors who experience treatment-induced menopause. Secondary outcomes include sexual functioning, sleep quality HF/NS frequency, psychological distress and overall HRQOL.

This trial has several notable strengths, including: (1) the randomized trial design; (2) the multicenter nature of the trial; (3) the comparison of both intervention groups with a waiting- list control group; (4) the use of intention-to-treat analysis; (5) the relatively long-term follow-up; and (6) the inclusion of a cost-effectiveness evaluation.

Several limitations of the trial should also be noted. First, it would be valuable to compare the Internet-based CBT groups with a face-to-face CBT group in order to compare compliance, experience and effectiveness. However, recruitment and follow up proved to be problematic in our previous group CBT trial (EVA-study) [44]. Also we consider it important to first establish the efficacy of the Internet-based CBT program. Second, we anticipate that both intervention groups (guided and self-management) will be effective, in comparison with the control group. The trial was powered based on the estimated effects of each of the two Internet-based CBT interventions in comparison to the control group. It may also be the case that one of the two CBT programs is more or less effective than the other. One would hope, given the additional costs involved, that the guided CBT program would be more efficacious than the self-management CBT program. However, if such differences exist, the magnitude of difference will likely be smaller than that expected between the CBT programs and the control group. In order to detect a smaller difference (effect size) when comparing the two variants of the CBT program, we would need a substantially larger sample size [88]. Unfortunately, our budget, both in terms of financial resources and time, does not allow us to increase the sample size. Nevertheless, within the limits of statistical power available to us, we will calculate between CBT group differences in both efficacy and cost-effectiveness outcomes. Finally, although women in the waiting-list control group will not be provided with any materials or program elements, they might look for other options themselves. However, we do not anticipate that this will take place in a structured or systematic way. In any case, at each assessment point, women are asked to report any activities that they may have undertaken to alleviate their menopausal symptoms.

In conclusion, given the rate and severity of treatment-induced menopausal symptoms in breast cancer survivors, there is a need for more easily accessible and efficient CBT interventions for these problems. If demonstrated to be efficacious and cost-effective, the availability of such a structured supportive intervention program will be a welcome addition to standard medical treatment offered to breast cancer survivors. It is anticipated that such a program will have direct benefit in terms of symptom relief and the improvement of patients’ HRQL, while making more efficient use of health care resources.

## Abbreviations

ANOVA: Analysis of variance
CBT: Cognitive behavioral therapy
CEA: Cost-effectiveness analysis
ES: Effect size
EuroQol-5D: EuroQol 5 dimension
FACT-ES: Functional assessment of cancer therapy-endocrine symptom scale
GSQS: Groningen sleep quality score
HADS: Hospital anxiety and depression scale
HF/NS: Hot flushes/night sweats
HFRS: Hot flush rating scale
HRQOL: Health related quality of life
HRT: Hormone replacement therapy
IRB: Institutional review board
ITT: Intention to treat
MOS-SS: Medical outcome study social support scale
PE: Physical exercise
PP: Per-protocol
QALY: Quality adjusted life year
RCT: Randomized controlled trial
REML: Restricted maximum likelihood
SAQ: Sexual activity questionnaire
SD: Standard deviation
SF-36: Short form 36
SF-6D: Short form 6 dimension
SSRI’s: Selective serotonin reuptake inhibitors
WLC: Waiting list control
